# Interaction of 4-Aminobutyrate (GABA) with the Tricarboxylic Acid Cycle in Plants Under Salinity Stress

**DOI:** 10.3390/plants15010123

**Published:** 2026-01-01

**Authors:** Edward J. Flaherty, Barry J. Shelp

**Affiliations:** Department of Plant Agriculture, University of Guelph, Guelph, ON N1G 2W1, Canada; eflahert@uoguelph.ca

**Keywords:** abiotic stress, 4-aminobutanal, 4-aminobutyrate, GABA, glutamate decarboxylase, metabolism, organic acids, polyamines, salinity, tricarboxylic acid cycle

## Abstract

The 4-aminobutyrate (GABA) shunt bypasses 2-oxoglutarate dehydrogenase and succinyl-CoA synthetase in the tricarboxylic acid cycle (TCAC) by diverting 2-oxoglutarate to glutamate and generating GABA via glutamate decarboxylase (GAD), whereas polyamine oxidation generates GABA directly from 4-aminobutanal. During salinity stress, the TCAC switches from a cyclic to a non-cyclic mode of operation probably due to the inhibition of two thiamine pyrophosphate-dependent enzymes, 2-oxoglutarate dehydrogenase and pyruvate dehydrogenase, and increases GAD activity via both transcriptional and post-transcriptional (i.e., elevated cytosolic Ca^2+^/calmodulin, H^+^ or glutamate) processes. Diversion of 2-oxoglutarate may occur via an increase in aminating glutamate dehydrogenase activity, due at least in part to the accumulation of ammonium, resulting from changes in protein synthesis and degradation. Inhibition of diamine oxidase activity by aminoguanidine suggests that polyamine oxidation contributes up to one-third of the salinity-regulated GABA level; however, *Arabidopsis thaliana* (L.) Heynh. GAD loss-of-function mutants suggest that polyamines account for less. The use of aminoguanidine and/or the GAD inhibitor, 3-mercaptopropionic acid, in combination with GAD or 4-aminobutanal dehydrogenase loss-of-function mutants, offers additional opportunities to understand if both GABA sources give rise to succinate, which can function to restore or partially restore TCAC activity during salinity stress.

## 1. Introduction

4-Aminobutyrate (GABA) is a ubiquitous, four-carbon, non-proteinogenic amino acid found in virtually all organisms. It was first identified in potato tuber (*Solanum tuberosum* L.) in 1949 and while it has been well studied over the years, the complete role of GABA in plants still remains unclear [1,2]. GABA is involved in various processes including carbon/nitrogen balance, pest defense, and protection against oxidative stress [2]. Discovery of GABA receptors provides strong evidence of GABA as a signaling molecule in plants [3]. Many of these roles are associated with the mitigation of stress, and a large body of literature describes GABA accumulation in plants in response to biotic and abiotic stresses [2,4,5]. GABA has a wide array of beneficial properties. For example, the application of exogenous GABA can mitigate abiotic stress-induced losses in plant growth, and genetic engineering of elevated GABA levels enhances resistance to biotic stresses [2].

Over the past two decades, increasing evidence has become available to suggest that organization of the tricarboxylic acid cycle (TCAC) in plants is dependent on metabolic and physiological demands of the cell [5,6,7,8]. Salinity is one form of stress that is known to limit plant growth and modify the regulation of the TCAC, thereby generating changes in cellular redox and energy balance [9,10]. Under such circumstances, the TCAC is likely to interact with a variety of metabolic pathways, including the GABA shunt (2-oxogluarate ⟶ glutamate ⟶ GABA ⟶ succinic semialdehyde ⟶ succinate). Therefore, salinity seems to be an appropriate stress model for studying the interaction between GABA and the TCAC. Here, we first provide an updated review of metabolic pathways for GABA synthesis from glutamate, polyamines and proline in the plant cell, including the transcriptional and post-transcriptional regulation of glutamate decarboxylase (GAD), the primary enzyme responsible for GABA synthesis. Second, we describe the impact of salinity stress on the TCAC and specific mechanisms that could activate GABA synthesis and restore respiration and energy production. Where appropriate, reference is made to other abiotic stresses. Particular attention has been paid to the experimental systems used by various researchers (e.g., plant organ, plant growing conditions, extent of salinity stress and biological system) and to biochemical versus physical effects, in order to identify gaps in our knowledge.

## 2. GABA Metabolism: The GABA Shunt

**Figure 1:**
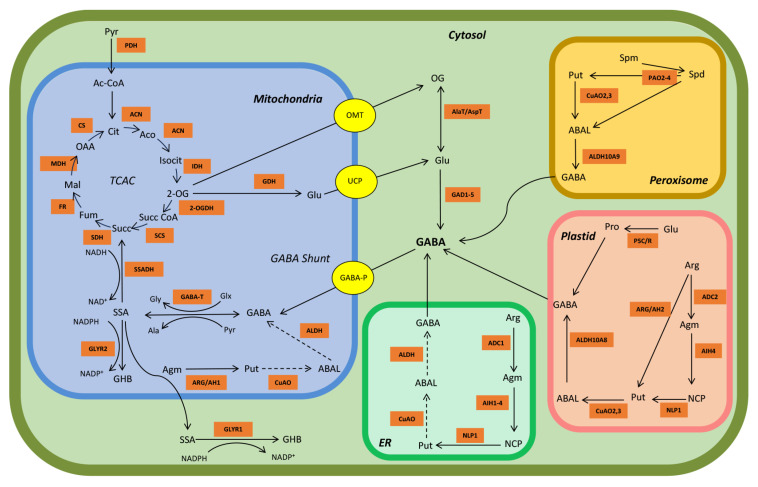
Model of GABA metabolism in arabidopsis. The orange squares represent key TCAC, GABA and polyamine metabolism enzymes. The yellow circles represent known transporters. Abbreviations: OG, 2-oxoglutarate; ABAL, 4-aminobutanal; Ac-CoA, acetyl-Coenzyme A; ACN, aconitase; Aco, *cis*-aconitate; ADC, arginine decarboxylase; Agm, agmatine; AIH, agmatine iminohydrolase; Ala, alanine; ALDH, aldehyde dehydrogenase; Arg, arginine; ARG/AH, arginase/agmatinase enzymes; Cit, citrate; CO_2_, carbon dioxide; CS, citrate synthase; CuOA, copper-containing amine oxidase; ER, endoplasmic reticulum; FR, fumarase; Fum, fumarate; GABA, γ-aminobutyrate; GABA-P; GABA permease; GABA-T, GABA transaminase; GAD, glutamate decarboxylase; GDH, glutamate dehydrogenase; GHB, γ-hydroxybutyrate; GLYR1/2, glyoxylate reductase (succinic semialdehyde reductase); Glu, glutamate; Glx, glyoxylate; Gly, glycine; AlaT/AspT, alanine transaminase (glutamate + pyruvate ↔ OG + alanine) or aspartate transaminase (glutamate oxaloacetate ↔ OG + aspartate); H^+^, hydrogen ion; IDH, isocitrate dehydrogenase; Isocit, isocitrate; Mal, malate; MDH, malate dehydrogenase; NADP^+^/NADPH, oxidized/reduced nicotinamide adenine dinucleotide phosphate; NCP, N-carbamoyl-putrescine; NLP1, N-carbamoylputrescine amidohydrolase; OAA, oxaloacetate; OG, 2-oxoglutarate; 2-OGDH, OG dehydrogenase OMT, 2-oxoglutarate/malate translocator; P5C/R, **Δ**^−1^-pyrroline-5-carboxylate synthase/**Δ**^−1^-pyrroline-5-carboxylate reductase; PAO, polyamine oxidase; PDH, pyruvate dehydrogenase; Pro, proline; Put, putrescine; Pyr, pyruvate; SCS, succinyl-CoA synthetase; SDH, succinate dehydrogenase; Spd, spermidine; Spm, spermine; SSA, succinic semialdehyde; SSADH, succinic semialdehyde dehydrogenase; Succ, succinate; Succ-CoA, succinyl-coenzyme A; UCP, uncoupling protein. Dotted arrows represent likely reactions for which no evidence yet exists.

GABA is an integral part of a conserved metabolic pathway known as the GABA shunt (Figure 1), which theoretically bypasses 2-oxoglutarate dehydrogenase and succinyl-CoA synthetase in the TCAC [5]. Typically, the GABA shunt is stimulated with the disruption of the TCAC upstream of succinate dehydrogenase [5,11,12]. For example, organellar thiamine pyrophosphate could become depleted in response to stress, thereby inhibiting 2-oxoglutarate dehydrogenase [13,14]. The GABA shunt begins with the diversion of 2-oxogluarate from the TCAC in the mitochondrion to produce glutamate [5]. Mitochondrial 2-oxoglutarate likely crosses the mitochondrial membrane via a 2-oxoglutarate/malate transporter and is converted into glutamate via an unknown transaminase activity in the cytosol [2,15]. Alternatively, 2-oxoglutarate may be converted into glutamate in the mitochondrion via glutamate dehydrogenase and then transported into the cytosol via an uncoupling protein; however, there is no direct evidence for this [2]. The next step of the GABA shunt is the irreversible α-decarboxylation of glutamate via cytosolic GAD to form GABA, which crosses the mitochondrial membrane via GABA permease and is converted into succinic semialdehyde via GABA transaminase using either pyruvate or glyoxylate as the amino acceptor [2,16,17]. The final step of the GABA shunt is catalyzed by succinic semialdehyde dehydrogenase, which irreversibly oxidizes succinic semialdehyde to succinate via an NAD^+^-dependent reaction [18]. Succinate can then be incorporated into the TCAC in the mitochondrion [5,19].

Alternatively, succinic semialdehyde can be reduced to 4-hydroxbutyrate (or γ-hydroxybutyrate) via the activity of NADPH-dependent glyoxylate/succinic semialdehyde reductases in the mitochondrion/plastid and cytosol, which is promoted by abiotic stress conditions and elevated NADH/NAD^+^ and NADPH/NADP^+^ ratios [2,20] (Figure 1). The accumulation of 4-hydroxbutyrate in response to drought, submergence, salinity, cold and heat in arabidopsis [*Arabidopsis thaliana* (L.) Heynh.] and submergence in tobacco (*Nicotiana tabacum* L.) is accompanied by the accumulation of GABA and alanine, and a decrease in glutamate [2]. This 4-hydroxbutyrate accumulation is proposed to be a coping mechanism to detoxify excess succinic semialdehyde [2]. It is noteworthy that the glyoxylate/succinic semialdehyde reductases have a much stronger affinity for glyoxylate than succinic semialdehyde (*K*_m_ = 2.2–34.2 µM vs. 870–8960 µM, respectively) [2].

Glutamate decarboxylase activity is a pyridoxal-5′-phosphate-dependent reaction that consumes a proton and produces CO_2_ as a by-product, so it may function to mitigate cytosolic acidification in plants [16,21,22,23]. Unlike animal or bacterial GADs, many plant GADs possess a 30–50 C-terminal amino acid domain that binds Ca^2+^/calmodulin complexes and activates GAD activity [24,25,26]. Thus, GABA production is also regulated via Ca^2+^/calmodulin. In vitro analysis of purified soybean (*Glycine max* L. Merr.), and recombinant petunia [*Petunia x hydrida* (Hooker) Vilm.], arabidopsis and apple (*Malus* x *domestica* Borkh.) GADs has shown that the pH optimum is approximately 5.8, with little activity at 7.0–7.5 in the absence of Ca^2+^/calmodulin [25,27,28]. However, GAD activity increases dramatically at pH 7.0–7.5 in the presence of Ca^2+^/calmodulin, though the activity is still less than that at pH 5.8. Transgenic tobacco plants overexpressing a mutant petunia or tobacco GAD lacking the autoinhibitory C-terminal domain (*GADΔC*) are unable to bind calmodulin, providing evidence for the importance of calmodulin binding for GAD activity in vivo [29,30,31]. Overexpression of *GADΔC* can lead to severe growth abnormalities, as well as elevated levels of GABA and decreased levels of glutamate. Together, these studies demonstrate that plant GAD is regulated by both Ca^2+^/calmodulin binding and pH.

*A. thaliana* has five known GAD isoforms, and three of these (*At*GAD1, 2 and 4) possess a Ca^2+^/calmodulin-binding domain [19,26]. The enzymatic activities of *At*GAD1,2 and soybean GAD at physiological pH are stimulated 35-, 13-, and four-fold, respectively, by Ca^2+^/calmodulin, but not by Ca^2+^ or calmodulin alone [25,32]. At pH 5.8, there is little to no effect of Ca^2+^/calmodulin binding [33]. The five arabidopsis *GAD* genes vary in their expression level and are tissue-specific [20]. At the transcriptional level, *AtGAD1* is predominantly expressed in roots, and does not appear to be stress-induced; however, low levels of *AtGAD1* are slightly upregulated in shoots in response to salinity or phosphorus deprivation [14,34]. *AtGAD2* is abundantly expressed throughout shoots and roots, and it is slightly upregulated in response to salinity, though this is likely a transient upregulation [20]. On the other hand, *AtGAD2* appears to be downregulated in response to hypoxia [35]. Expression of *AtGAD4* is thought to be upregulated in both roots and shoots by salinity, as well as other stresses, whereas expression is minimal in the absence of stress [32,34,35,36,37]. However, expression of *AtGAD4* has also been shown to be unaffected in both root and shoot tissue by salinity stress [38]. Under both stress and non-stress conditions, *AtGAD3,5* are only weakly expressed if at all, in vegetative organs, and apparently lack the Ca^2+^/calmodulin binding domain [20,26].

GAD inhibitors and loss-of-function plants offer insight into the roles and activities of the various GAD isoforms. For example, GAD activity and GABA production are decreased in arabidopsis pollen tubes grown in tissue culture medium containing 1 mM 3-mercaptopropionic acid, and respiratory O_2_ production is decreased in disks of salt-stressed wheat leaves incubated in 10 mM mercaptopropionic acid [39,40]. The *atgad1* mutant has only 14% of the constitutive root GABA level in wild-type plants, and heat-stress-induced GABA accumulation is prevented [41], suggesting that GAD1 is crucial for maintaining root GABA levels under both stress and non-stress conditions. Similarly, GABA levels in leaves and roots are less in *atgad1/2* than that in wild type under both control and saline conditions, though there is also evidence that root GABA is unaffected by salinity [34,38,42] (Table 1). Under drought conditions, GABA does not accumulate in leaves of *atgad2* [43]. Notably, the GABA level in *atgad4* shoot is similar to that in wild type under non-stress conditions but is induced by one- to two-fold after 2 d treatment with 150 mM NaCl [34]. The constitutive shoot level of GABA of *atgad1/2* is less than that in wild-type plants, but the level induced by 2 d of 150 mM salt is less than the corresponding levels in wild-type and *atgad4* plants, suggesting that *At*GAD1 and *At*GAD2 are more important than *At*GAD4 for salinity-induced GABA production, though *At*GAD4 does contribute marginally. A second study demonstrated that roots of 4-wk-old *atgad1/2* plants have similar constitutive GABA levels as wild type, and while the wild type accumulates 75% more GABA after 15 min with 100 mM NaCl, the mutant accumulates 40% [42]. However, in another study, 4-wk-old *gad1/2* seedings have markedly lower constitutive GABA levels than wild-type plants, and when subjected to 150 mM NaCl for 2 wk, do not accumulate GABA [38]. These studies demonstrate uncertainty about the contribution of *At*GAD4 to both constitutive and stress-induced GABA accumulation.

## 3. Alternative GABA Biosynthetic Pathways

While the decarboxylation of glutamate by GAD is the main biosynthetic reaction for GABA, polyamines and theoretically proline are also precursors for GABA biosynthesis, and their importance is likely to be enhanced by abiotic and biotic stresses [44,45]. Multiple biosynthetic routes exist for polyamines (Figure 1). In the peroxisome, the oxidation of the secondary and tertiary polyamines spermidine and spermine, respectively, by polyamine oxidase2,3 and FAD-dependent polyamine oxidase2–4, respectively, leads to the primary polyamine putrescine and 4-aminobutanal [44]. Alternatively, putrescine may be synthesized in the plastid or endoplasmic reticulum in a three-step process from arginine to agmatine to N-carbamoylputrescine by the enzymes arginine decarboxylase, agmatine imidohydrolase, and N-carbamoylputrescine amidohydrolase [46,47]. Putrescine can also be directly synthesized from arginine and agmatine by the enzymes arginase/agmatinase in the plastid and mitochondrion/endoplasmic reticulum, respectively, but to date, there is no evidence for a plastidial or mitochondrial copper amine oxidase (CuAO) for the conversion of putrescine to GABA [47,48,49].

Putrescine can be converted to 4-aminobutanal by a peroxisomal CuAO [2,44,50] (Figure 1). Roots of 2-wk-old soybean plants treated with 50–150 mM NaCl for 6 d exhibit marked decreases in spermine, spermidine and putrescine, and increases in diamine oxidase activity and GABA level. Also, the diamine oxidase inhibitor aminoguanidine (1 mM) strongly decreases diamine oxidase activity, but increases polyamine accumulation and decreases GABA [51]. Under salt stress, aminoguanidine-treated soybean roots accumulate approximately 39% less GABA than control plants, suggesting that 39% of the total GABA is derived from polyamine oxidation via diamine oxidase, while the remaining 61% is derived directly from glutamate via GAD activity. Leaves of 2-wk-old tea [*Camellia sinensis* (L.) Kuntze] plants exhibit a 20-fold increase in GABA after 11 h of anoxia, relative to the untreated control, as well as a 50-fold increase in diamine oxidase activity, whereas GAD activity exhibits a transient one-fold increase at 3–4 h, followed by a return to the control level at 11 h [52]. The expression of *CsGAD1* and *CsGAD2* increases by five- to 10-fold at 11 h, relative to the 0 h control, whereas the levels of putrescine and spermidine increase by less than one-fold. The diamine oxidase activity in anoxia-treated leaves sprayed with 5 mM aminoguanidine is completely inhibited and GABA decreases by 30%. Together, these results suggest that approximately one-third of the GABA generated under salinity or anoxia could be derived from polyamines.

4-Aminobutanal is oxidized to GABA via NAD^+^-dependent aminoaldehyde dehydrogenase (AMADH) [44,53,54] (Figure 1). Two *AMADH* genes, designated as *ALDH10A9* (*AMADH1*) and *ALDH10A8* (*AMADH2*), appear to be localized in peroxisome and plastid, respectively [53,54]. Both *AtALDH10A8* and *AtALDH10A9* are constitutively expressed in arabidopsis and may be weakly upregulated in response to salinity and dehydration [55,56]. Zarei et al. [53,54] demonstrated that recombinant apple and arabidopsis ALDH10A8 and ALDH10A9 can utilize 4-aminobutanal and 3-aminopropanal [(apparent *K*_m_s = 25–160 and 8.6–14 μM for 4-aminobutanal and 3-aminopropanal, respectively) and (apparent *K*_m_s = 85–460 and 16–17 μM for 4-aminobutanal and 3-aminopropanal, respectively)] as substrates, catalyzing the synthesis of both GABA and β-alanine. Furthermore, 11-d old, tissue-cultured *ataldh10A8* and *ataldh10A9* seedlings grown for 4 d with 150 mM NaCl have a similar phenotype and GABA level as control wild-type plants; however, GABA accumulation is decreased by approximately 30–50%, compared to treated wild-type plants, and necrotic lesions, purpling of leaves and inhibition of root growth are evident. Jacques et al. [57] reported that recombinant *At*ALDH108,9 can effectively use trimethylaminobutyraldehyde, as well as 4-aminobutanal and 3-aminopropanal, leading to the synthesis of 4-butyrobetaine. Tissue-culture-grown, 11-day-old *ataldh10A8/9* seedlings receiving 4 d of 150 mM NaCl, and soil-grown, 5-wk-old *ataldh10A8/9* plants receiving 1 wk of 150 mM NaCl do not show a difference in GABA level from untreated wild-type plants, though stem length is dramatically reduced. The promiscuous activities of the ALDH10As may be important determinants of crosstalk among metabolic pathways during stress [5].

Exogenous putrescine, drought and anoxia increase the GABA level in tea plants, and putrescine increases the expression of peroxisomal *CsCuAO1,3*, but not plastidial *CsAMADH1* and *CsGAD1–3* [58]. Recombinant *Cs*AMADH1 has a *K*_m_ for 4-aminobutanal of 21.9 mM, which is one to two orders of magnitude greater than the values reported above and possibly due to a failure to consider the impact of substrate inhibition, which is characteristic of this family of proteins, on the kinetic properties [53,54,57]. Nevertheless, exogenous GABA enhances drought tolerance and increases the expression of *CsCuAO1* and *CsAMADH1* in roots, *CsCuAO1* and *CsGAD1* in stems, and *CsGAD1,2* in leaves [58]. Arabidopsis lines overexpressing *CsCuAO1* and *CsAMADH1* are more drought resistant than wild type, whereas suppression of *CsCuAO1* or *CsAMADH1* in tea plants increases drought sensitivity. Co-overexpression of *CsCuAO1* and *CsAMADH1* increases GABA accumulation both in an *Agrobacterium*-mediated *Nicotiana benthamiana* Domin. transient assay and in transgenic arabidopsis plants [59]. The results of this study suggest that CsCuAO1 and CsAMADH1 are involved in the response to drought stress and that further research is required on the contribution of polyamines to GABA production during salinity.

Proline is primarily derived in the plastid from two successive steps from the precursor glutamate, by the enzymes **Δ**^−1^-pyrroline-5-carboxylate synthase and **Δ**^−1^-pyrroline-5-carboxylate reductase [60]. Proline can react with stress-produced hydroxy radicals by H-abstraction of the amine group, with the spontaneous decarboxylation of proline, leading to the formation of pyrrolidin1-yl, which can then be converted to **Δ**^−1^-pyrroline, the substrate for the enzyme **Δ**^−1^-pyrroline dehydrogenase (believed to be the same enzyme as 4-aminobutanal dehydrogenase), resulting in the generation of GABA [44,61]. Since there is no direct evidence for the contribution of proline to GABA production in planta, the significance of this reaction is uncertain.

## 4. Effect of Salinity Stress on the Tricarboxylic Acid Cycle

The TCAC is generally considered to function as a cyclic sequence of oxidative reactions which ultimately generate the reducing equivalents NADH and FADH_2_ to drive ATP production in the mitochondrial electron transport chain (Figure 1). However, the physiological and metabolic demands of the plant can also result in non-cyclic flux [5,6,7,8]. The demand for ATP itself may act as a switch between cyclic and non-cyclic flux. As the demand for ATP increases, carbon flow through the TCAC increases, whereas when demand is low, TCAC-carbon may be re-allocated outside of the TCAC to other metabolic processes [62]. For example, plant productivity, including photosynthesis and growth, decreases under salinity stress, while carbon is re-allocated to respiration [40,63,64]. The TCAC also interacts with other metabolic networks including, but not limited to amino acid metabolism, photosynthesis and photorespiration [65,66,67]. Individual TCAC metabolites can also have roles in other pathways. Salt stress generally leads to increases in amino acid levels, either through elevated protein degradation or *de novo* synthesis, as well as alterations in organic acid levels, which provides unambiguous support for a salinity-induced switch from a cyclic to a non-cyclic mode of operation for the TCAC [10,40,68] (Table 2).

The shoots of 4-wk-old, soil-grown arabidopsis wild-type plants treated with 150 mM NaCl accumulate less fumarate, oxaloacetate, malate and citrate than control plants, whereas succinate is unaffected [38]. In contrast, the *gad1/2* mutant accumulates more oxaloacetate, malate and citrate, while succinate and fumarate levels are unaffected, compared to the wild type. It is noteworthy that this study measured TCAC metabolites in shoots, but not roots. A 4-d treatment with 150 mM NaCl decreases the 2-oxoglutarate level and increases the succinate level in roots of the *atgaba-t* (*atpop2*) mutant, whereas a 1-d treatment with 150 mM NaCl decreases succinate and increases malate in roots; notably, the metabolic profiles of *atgaba-t* and wild-type shoots are unaffected by the salinity stress [74,75]. While these two studies offer insights into the effect of salinity stress on the TCAC, tissue culture-grown plantlets were used. Since these plants do not readily transpire, the plants would be in an unnatural physiological state and therefore, may not properly activate stress-induced metabolic changes.

Che-Othman et al. [40] recently demonstrated that the third leaf of hydroponically grown wheat plants treated with 150 mM salt for 11 d have elevated levels of 2-oxogluarate and succinate compared to control plants, and decreases in fumarate, malate, citrate and aconitate (Table 2). The increases in 2-oxogluarate and succinate are accompanied by a decrease in abundance of 2-oxogluarate dehydrogenase subunits and an increase in 2-oxoglutarate/malate transporter subunits, indicating that 2-oxoglutarate may be transported out of mitochondria for use outside of the TCAC. Alternatively, the increase in 2-oxoglutarate may be due to increases in the activity of isocitrate dehydrogenase or glutamate dehydrogenase (GDH). The greatest decreases in organic acids in response to salinity stress are aconitate and citrate (<1% and <12% control values, respectively). Since these two metabolites are directly downstream of pyruvate, Che-Othman et al. [40] suggested that physical changes in the pyruvate dehydrogenase complex could account for the result. Subsequently, Kumari et al. [76] reported that leaves of 15-d-old wheat plants subjected to 100 mM NaCl for 30 d have decreased activities of pyruvate dehydrogenase (a thiamine pyrophosphate-dependent enzyme), citrate synthase, NAD^+^-isocitrate dehydrogenases, succinate dehydrogenase and malate dehydrogenase, compared to the untreated control. While it is unknown how the salinity stress inhibits the enzymatic actions of the TCAC, salinity clearly modifies the regulation of the TCAC, resulting in non-cyclic flux and changes in energy balance [5,6,7]. Overall, plant species, treatment level and duration, growth conditions, plant age and organ type may contribute to the differential response of salt-induced changes in organic acid levels in plants [77,78].

## 5. Stress-Induced Activation of GABA Metabolism Restores Energy Production and Respiration

**Table 3:** Salinity stress increases GABA levels in various plant species.

Plant Species	Growth Condition	Organ/ Tissue	NaCl Treatment	Control [GABA]	Salt [GABA]	Fold Stimulation	Reference
Tomato (*Solanum lycopersicum* L.)	Hydroponics	Leaf	175 mM, 2 d	~28 μmol g^−1^ FM	~42 μmol g^−1^ FM	0.5	[79]
			175 mM, 4 d	~23 μmol g^−1^ FM	~30 μmol g^−1^ FM	0.3	
White clover (*Trifolium repens* L.)	Tissue culture	Seedling	100 mM, 7 d	~0.32 μmol g^−1^ DM	~0.24 μmol g^−1^ DM	~0.3	[80]
Legume shrub (*Caragana intermedia* L.)	Sand	Root	300 mM, 2 d	~5 nmol g^−1^ FM	~35 nmol g^−1^ FM	~6	[81]
Arabidopsis (*Arabidopsis thaliana* [L.] Heynh.)	Tissue culture	Shoot	150 mM, 4 d	0.7 μmol g^−1^ DM	11 μmol g^−1^ DM	15	[75]
		Root		7.5 μmol g^−1^ DM	9.9 μmol g^−1^ DM	0.3	
	Hydroponics	Root	150 mM, 1 d	6.7 μmol g^−1^ DM	6.9 μmol g^−1^ DM	0.03	[74]
	Soil	Shoot	150 mM, 14 d	0.02 μmol g^−1^ FM	0.040 μmol g^−1^ FM	1	[38]
		Root		0.4 μmol g^−1^ FM	1.3 μmol g^−1^ FM	2.3	
	Tissue culture	Root	100 mM, 15 min	~0.13 μmol g^−1^ FM	0.6 μmol g^−1^ FM	~3.6	[42]
	Soil	Shoot	150 mM, 2 d	~0.02 μmol g^−1^ FM	~0.07 μmol g^−1^ FM	~2.5	[34]
Rice (*Oryza sativa* L.)	Tissue culture	Leaf	150 mM, 7 d	34 μmol g^−1^ DM	46 μmol g^−1^ DM	0.35	[82]
Wheat (*Triticum durum* Desf.)	Hydroponics	Shoot	100 mM, 10 d, 350 μmol m^−2^ s^−1^ PAR	~1 μmol g^−1^ DM	~1.5 μmol g^−1^ DM	1.5	[83]
			100 mM, 10 d, 900 μmol m^−2^ s^−1^ PAR	~8 μmol g^−1^ DM	~46 μmol g^−1^ DM	6	
Corn (*Zea mays* L.)	Hydroponics	Leaf	150 mM; 12, 36 and 60 h	~252, 290, 290 μmol g^−1^ FM	~290, 533, 436 μmol g^−1^ FM	0.13, 0.84, 0.5	[71]

Under normal physiological conditions, plant tissue GABA levels generally range from 0.03 to 2 μmol g^−1^ fresh mass); however, both biotic (e.g., viral, bacterial and fungal infection) and abiotic stresses (e.g., salinity, hypoxia, waterlogging, cold, heat and ultra-violet radiation, alone or in combination) can increase GABA accumulation by several-fold in various plant species, organs and cell types [4,5,20]. GABA levels generally increase in response to salt stress, with the fold stimulation varying widely with method of cultivation, duration of stress exposure, and plant species (Table 3). Notably, GABA does not necessarily accumulate because the carbon flux through the GABA shunt into the TCAC or into 4-hydroxybutyrate could increase [2,75,84,85].

Recent research demonstrated that wheat plants subjected to 150 mM NaCl for 11 d have lower photosynthetic rates and biomass than control plants, while respiration rates increase [40]. Salt-treated plants generally have elevated levels of glutamate and GABA compared to control plants, and this is accompanied by increases in *GAD*, *GABA transaminase* and *succinic semialdehyde dehydrogenase* expression, and in GAD activity. In another study, leaves of wheat plants subjected to 100 mM NaCl for 30 d have elevated activities of GAD, GABA transaminase, succinic semialdehyde dehydrogenase and GDH, as well as GABA and glutamate levels [76]. Similarly, arabidopsis plantlets treated with 150 mM NaCl for 8 d exhibit a four-fold increase in GABA levels compared to control plantlets, as well as elevated GAD and GABA transaminase activities [75]. These results suggest that the GABA shunt is activated when TCAC activity is diminished during salinity stress (see Section 4). Thus, the GABA shunt pathway could provide an alternative pathway for the production of TCAC intermediates, as well as ATP, NADH and FADH_2_, thereby alleviating oxidative damage and promoting stress tolerance [2,40].

Glutamate dehydrogenase could provide an important link between the TCAC and the GABA shunt during salinity stress, as it is capable of assimilating ammonia into glutamate, as well as deaminating glutamate into 2-oxoglutarate and ammonia [86]. Aminating GDH activity increases in salt-tolerant rice (*Oryza sativa* L.) cultivars with increasing salt stress, whereas it decreases in salt-sensitive cultivars [87]. The aminating GDH activity is high in roots of pea (*Pisum sativum* L.), an ammonium-tolerant plant [88]. Skopelitis et al. [89] demonstrated that NaCl treatment induces reactive oxygen species, tissue ammonia (from 0.4–0.6 to 1.4–1.6 μmol g^−1^ fresh mass in stems and leaves), expression of tobacco *gdh-NAD;A1* encoding the α-subunit of GDH, assembly of the anionic GDH isoenzymes, in vitro GDH aminating activity in tissues, in vivo aminating GDH activity, and upregulation of NAD^+^- and NADP^+^-dependent *isocitrate dehydrogenase* genes. Exogenous ammonium (10 mM) also mimics the effects of salinity in induction of *gdh-NAD;A1* expression in tobacco and grape (*Vitis vinifera* L.) suspension cells. Together, these results suggest that salinity induces α-GDH subunit expression, and the anionic GDHs assimilate ammonia, acting as antistress enzymes in ammonia detoxification.

Grzechowiak et al. [90] reported that the *K*_M_ 2-oxoglutarate for NAD(H)-dependent *At*GDH1 and *At*GDH2 is 0.20 mM and 0.61 mM, respectively, at a non-saturating ammonium concentration of 10 mM, giving a catalytic efficiency of 0.14 and 0.023 s^−1^ μM^−1^, whereas the *K*_M_ glutamate is 0.58 and 0.98 mM, respectively, giving a catalytic efficiency of 0.035 and 0.033 s^−1^ μM^−1^. Notably, the enzymes are not saturated at 10 mM ammonium. These *K*_M_ 2-oxoglutarate values are similar to those reported for three corn (*Zea mays* L.) leaf GDH isoforms (0.22–0.60 mM) [91]. These results suggest that reductive amination is the preferred catalytic direction, at least for *At*GDH1 under the in vitro conditions tested. Yamaya et al. [92] estimated the physiological concentration of ammonium in corn leaf mitochondria to be 5–10 mM and demonstrated that mitochondria can tolerate these relatively high concentrations of ammonium. A high concentration of ammonium either provided exogenously or as the result of protein hydrolysis generally increases GDH activity, and this is often associated with changes in the isoenzyme profile [93]. The presence of two different GDH subunits (α or β), which can assemble into homohexamers and heterohexamers, can affect glutamate accumulation; however, the mechanism regulating the heterohexamer composition is unclear [94,95,96,97]. Notably, the conversion of 2-oxoglutarate to glutamate at physiological pH consumes a proton; therefore, the aminating reaction of GDH could theoretically play a role in pH regulation.

Recently, Eprintsev et al. [98] demonstrated that incubation of 2-wk-old, detached shoots of corn in 150 mM NaCl changes the expression of *GDH1* and *GDH2* genes and enhances leaf GDH activity by three-fold up to 24 h. This is accompanied by decreases in 2-oxoglutarate dehydrogenase activity and *2-oxoglutarate dehydrogenase1,2* expression after 6–8 h. These results were interpreted as support for the assembly of the native GDH molecule having a different subunit composition and greater affinity for 2-oxoglutarate, thereby diverting 2-oxoglutarate flux from the TCAC to the GABA shunt. Decarboxylation by the TCAC and pyruvate dehydrogenase decreases in illuminated leaves by up to 80% and 30%, respectively, compared to dark respiration [99]. Interestingly, this is associated with marked decreases in 2-oxoglutarate oxidation and GDH activity, so that glutamate for decarboxylation by GAD is likely to be derived via the glutamine synthetase/glutamate synthase cycle [100]. Consequently, GABA could maintain the TCAC and mitochondrial electron transport in an active photosynthetic cell with decreased operation of the TCAC resulting from the high level of ATP and reducing equivalents, even though the glutamate would be derived from 2-oxoglutarate outside the mitochondrion. This could have implications for interaction of GABA and the TCAC in shoots versus roots of plants subjected to salinity stress. Interestingly, Fontaine et al. [101] investigated the metabolic response of an *atgdh1/2/3* triple mutant to 7 d of continuous darkness, which should simulate carbon starvation and elevate ammonium levels. They concluded that the main physiological function of the NADH-GDH is to provide 2-oxoglutarate to the TCAC, mainly through processes operating in the root, even though ammonium decreases in leaves, 2-oxoglutarate decreases in both leaves and roots, and GABA increases in both leaves and roots when activity of the glutamine synthetase/glutamate synthase cycle should be minimal. Since both the TCAC and the glutamine synthetase/glutamate synthase cycle would be suppressed by salinity, regardless of the light condition, it is tempting to speculate that 2-oxoglutarate is diverted from the TCAC via an increase in aminating GDH activity, due at least in part to the accumulation of ammonium resulting from changes in protein synthesis and degradation. Overall, these results suggest that GAD can utilize glutamate from multiple sources.

At least two biochemical mechanisms, acidic pH and Ca^2+^/calmodulin, exist to stimulate GAD activity in vitro [25,27,28,33]. Data are available to support the acidic pH stimulation of plant GADs in cell-suspension cultures of carrot (*Daucus carota* L.) and isolated asparagus (*Asparagus sprengeri* Regel) mesophyll cells in response to ammonium assimilation, hypoxia and acid loading, which reduce cytosolic pH by 0.2–0.6 units [22,23]. Plant cytosolic pH ranges from 7.1 to 7.5 and salt-induced acidification of the cytosol ranges from 0.4 to 1.3 pH units [102,103]. Furthermore, increasing salinity results in Ca^2+^ influx into plant cells, thereby elevating cytosolic free Ca^2+^ that can bind calmodulin as a secondary messenger for adaptive signaling [4,104,105]. Based upon the kinetics of GABA accumulation, it is possible that stress-specific GAD stimulation can be divided into two phases: Ca^2+^/calmodulin may act as a rapid or initial response to stress and/or a response to a mild or transient stress; and acidic pH may act in a Ca^2+^/calmodulin-independent manner with extended duration and/or severity of the stress [4,106]. It is not trivial to discern between these two options [106,107]. Also, stress-induced stimulation of GAD activity could be a response to an elevated level of glutamate, resulting from changes in protein synthesis or degradation and ammonium assimilation [94,108].

Transcription could also increase GAD activity in response to salinity. However, expression of *AtGAD1,2* is only slightly affected, if at all, by salt concentration up to 150 mM NaCl and any increase is often temporary [20,34,75] In contrast, the expression of *AtGAD4* is markedly increased, though it is still only at a low level relative to *AtGAD1,2*. Recently, Eprintsev et al. [98] reported that a maize leaf *GAD* gene (LOC100284) is stimulated by more than 20-fold within 12 h of exposure to 150 mM NaCl, then it returns to the control level by 24 h. Corn probably contains five *GAD* paralogs [26], so the significance of this particular *GAD* gene to overall *GAD* expression and activity in the leaf is unclear. Nevertheless, these studies emphasize the importance of monitoring *GAD* expression as a function of time after exposure to salinity.

Extensive evidence exists in the literature for stress-regulated stimulation of polyamine catabolism and GABA formation [44,109]. For example, aminoguanidine has been used to demonstrate that polyamine degradation contributes 25–39% of the GABA generated in: roots of 2-wk-old soybean seedlings under salinity; germinating fava bean (*Vicia faba* L.) under hypoxia; and tea leaves under anoxia [51,52,110]. Roots of 9 d old seedlings of *ataldh10a8* and *ataldh10a9* mutants accumulate 50% less GABA than wild-type plants and are more sensitive to salinity (after receiving 150 mM NaCl for 4 d) [62]. Similarly, Jacques et al. [57] showed that the GABA level in salt-stressed 15 d old seedlings (after 4 d on 50 mM NaCl) or leaves of 6-wk-old plants (after 1 wk of 150 mM NaCl) of the *ataldh10a8/9* mutant was approximately 50% of the levels in corresponding wild-type plants, though the data were not significant, perhaps because only three biological replicates were used. Metabolite analysis suggested that GABA accumulated by tea leaves treated with a combination of drought and heat stress is derived from both polyamine degradation and the GABA shunt [111].

Other research suggested that GABA formation from polyamines or proline can account for only a minor portion of the salinity-regulated generation of GABA in wild-type plants. For example, the shoot GABA level of *atgad1/2* plants is approximately 4–15% of that in corresponding wild-type plants under drought or salinity stress [38,42,112]. Leaves of wheat plants subjected to 150 mM NaCl for 11 d have lower photosynthetic rates and biomass than control plants, while respiration rates increase [40]. The salt-treated leaves generally have lower levels of fumarate, malate, citrate and aconitate and elevated levels of glutamate and GABA, and this may be accompanied by increases in GAD activity. Similarly, arabidopsis plantlets treated with 150 mM NaCl for 8 d exhibit a four-fold increase in GABA, compared to control plantlets, as well as elevated GAD activity [75]. Overall, these results suggest that TCAC activity is diminished during salinity stress, while GABA metabolism is activated. Further research is required to establish the relative importance of the GABA shunt versus polyamine degradation in sustaining TCAC activity during salinity stress. Nevertheless, it is clear that both pathways can lead to the generation of succinate, as well as ATP, NADH and FADH_2_, thereby alleviating oxidative damage and promoting stress tolerance [2,40].

## 6. Concluding Remarks

Salinity stress increases respiration and changes the levels of many metabolites in plants, including those associated with the GABA shunt, polyamine oxidation and the TCAC. The GABA shunt can theoretically bypass the 2-oxoglutarate dehydrogenase and succinyl-CoA synthetase reactions in the TCAC by diverting 2-oxoglutarate to glutamate and the GAD-mediated generation of GABA (i.e., the GABA shunt), whereas polyamine oxidation generates GABA directly from 4-aminobutanal. When the TCAC switches from a cyclic to a non-cyclic mode of operation during salinity stress, both GABA sources can give rise to succinate and function to restore or partially restore TCAC activity. Knowledge of the exact mechanisms (biochemical versus physical, primary versus secondary) responsible for the metabolic changes is incomplete, at least in part because of differences in experimental system among studies (e.g., plant organ, plant cultivation, extent of salinity stress and biological system). Nevertheless, available evidence suggests that salinity stress can inhibit both 2-oxoglutarate dehydrogenase and pyruvate dehydrogenase activities, possibly due to the depletion of thiamine pyrophosphate, and increase GAD activity via both transcriptional and post-transcriptional processes (i.e., elevated cytosolic Ca^2+^/calmodulin, H^+^ or glutamate), depending on the GAD paralog/GAD isoform under consideration. The fate of 2-oxoglutarate derived from isocitrate dehydrogenase in the TCAC appears to differ between darkness and light. In darkness, it is directly converted into succinyl-CoA, whereas in light it is diverted to the chloroplast for conversion to glutamate. With salinity, it is tempting to speculate that the diversion of 2-oxoglutarate occurs via an increase in aminating GDH activity, due at least in part to the accumulation of ammonium resulting from changes in protein synthesis and degradation. Inhibitor evidence suggests that polyamine oxidation can contribute up to one-third of the GABA generated during salinity stress; however, GAD loss-of-function mutants suggest that polyamines and proline account for even less. The sole or combined use of chemical inhibitors such as aminoguanidine and 3-mercaptopropionic acid and loss-of-function mutants such as *atgad1/2/4* and *ataldh10a8/9* offer additional opportunities to understand the interaction between GABA metabolism and the TCAC during salinity stress.

## Figures and Tables

**Table 1:** Salinity-activated increases in GABA can be markedly reduced in *At*GAD loss-of-function mutants.

Genotype	Growth Condition	Organ	NaCl Treatment	Control [GABA] ^2^	Salt [GABA] ^2^	Fold Stimulation	Reference
Wt ^1^	Soil	Shoot	150 mM, 2 d	~0.018	~0.063	~2.5	[34]
*gad4*				~0.020	~0.038–0.050	~0.9–1.5	
*gad1/2*				~0.005	~0.007–0.018	~0.4–2.6	
Wt	Soil	Shoot	150 mM, 14 d	~0.02	~0.04	~1	[38]
Wt		Root		~0.4	~1.3	~2.3	
*gad1/2*		Shoot		n.d. ^3^	n.d.	-	
*gad1/2*		Root		~0.0125	~0.0125	0	
Wt	Tissue culture	Root	100 mM, 15 min	~0.39	~0.69	~0.77	[42]
*gad1/2*				0.33	~0.45	~0.36	

^1^, wild type; ^2^, μmol g^−1^ fresh mass; ^3^, not detectable.

**Table 2:** Salinity-induced changes in relative levels of organic acids signal a switch from a cyclic to a non-cyclic mode of TCAC operation in various plant species.

Plant Species	Growth Condition	Organ/ Tissue	NaCl Treatment	Metabolite Increase	Metabolite Decrease	Reference
Wheat (*Triticum aestivum* L.)	Hydroponics	Leaf	150 mM, 3 d	OG, Succ	Fum, Mal, Cit, Aco	[40]
Cucumber (*Cucumis sativus* L.)	Soil	Leaf	75 mM, 7 d	-	Pyr, Cit, Succ, Mal	[69]
Corn (*Zea mays* L.)	Hydroponics	Root	100 mM, 4 d	-	Mal, Fum, Cit, OG	[70]
		Leaf	150 mM; 12, 36, 60 h	-	Mal, Cit, Succ, OG	[71]
Barley (*Hordeum vulgare* L.)	Hydroponics	Root	300 mM, 21 d	Cit, Isocit	Fum, OG	[72]
		Shoot		-	Cit, Fum, OG, Mal, Succ	
Tobacco (*Nicotiana tabacum* L.)	Tissue culture	Shoot	500 mM, 3 d	Succ	Mal, Fum	[73]
Arabidopsis (*Arabidopsis thaliana* [L.] Heynh.)	Soil	Shoot	150 mM, 14 d	-	Fum, OAA, Mal, Cit	[38]

Abbreviations: Aco, aconitate; Cit, citrate; Fum, fumarate; Isocit, isocitrate; Mal, malate; OAA, oxaloacetate; OG, 2-oxoglutarate; Pyr, pyruvate; Succ, succinate.

## Data Availability

No new data were created or analyzed in this study.

## Abbreviations

The following abbreviations are used in the main text of this manuscript:

GABA	4-Aminobutyrate
GAD	Glutamate decarboxylase
GDH	Glutamate dehydrogenase
TCAC	Tricarboxylic aid cycle
*K*_m_	Michaelis-Menten constant
AO	Amine oxidase
AMADH	Aminoaldehyde dehydrogenase
ALDH	Aldehyde dehydrogenase
